# Adrenalectomy abolishes hypergravity-induced gastric acid hyposecretion

**DOI:** 10.18632/oncotarget.15408

**Published:** 2017-02-16

**Authors:** Kiyong Na, Hyun-Soo Kim

**Affiliations:** ^1^ Department of Pathology, Severance Hospital, Yonsei University College of Medicine, Seoul, Republic of Korea

**Keywords:** adrenalectomy, hypergravity, gastric acid, gastrin, rat, Pathology Section

## Abstract

Jet fighter pilots experience high gravitational acceleration forces in the cephalocaudal direction (+Gz), causing severe stress. Stress affects different physiological functions of the gastrointestinal tract. Although the effects of exposure to hypergravity on cardiovascular and cerebral functions have been the subject of numerous studies, crucial information regarding potential pathophysiological alterations following hypergravity exposure in the gastrointestinal tract is lacking. We recently documented a significant decrease in gastric secretory activity in rats after acute exposure to hypergravity. In the present study, we investigated the effects of adrenalectomy on gastric acid secretion and plasma gastrin level in hypergravity-exposed rats. Male Sprague-Dawley rats were adrenalectomized and exposed to +10Gz three times for 3 min. Gastric juice and blood samples were collected, and the volume and total acidity of gastric juice and plasma level of gastrin were measured. Consistent with our previous data, acute exposure to +10Gz significantly altered the gastric juice parameters in the sham-operated rats. The volume (*P* < 0.001) and acidity (*P* < 0.001) of gastric juice in the hypergravity-exposed rats were significantly lower than those in the nonexposed rats. In contrast, in adrenalectomized rats, the differences in the gastric juice volume (*P* = 0.712) and acidity (*P* = 0.279) were not statistically significant between the hypergravity-exposed and nonexposed rats. We demonstrated that adrenalectomy abolished hypergravity-induced gastric acid hyposecretion, but did not influence gastrin release. These findings suggest that the adrenal glands are required for hypergravity-induced gastric acid hyposecretion.

## INTRODUCTION

Gastric acid secretion is regulated by both the autonomic nervous system and hormones. The parasympathetic nervous system and gastrin stimulate the parietal cells to produce gastric acid, both directly acting on the parietal cells and indirectly through the stimulation of histamine secretion from the enterochromaffin-like cells [1]. The highly acidic environment in the stomach causes proteins from food to lose their characteristic folded structure. This exposes the peptide bonds of the proteins. The chief cells of the stomach secrete the enzyme for protein breakdown, inactive pepsinogen [2]. Gastric acid activates pepsinogen into pepsin, which then helps digestion by breaking the bonds linking the amino acids in a process known as proteolysis. In addition, many microorganisms have their growth inhibited by such an acidic environment, which is helpful for preventing infection [1–3].

In fighter jets, pilots experience exceptionally high gravitational acceleration (G) forces in the cephalocaudal direction (+Gz). With the development of modern, lightweight, high-thrust aircraft, G limits are mainly determined by the physiology of the pilot, rather than the performance or structural limitations of aircrafts. Military fighter pilots are exposed to sustained and repeated hypergravity to levels as high as +9Gz. Marked alterations in the autonomic nervous system occur from the baroreceptor stimulation resulting from performing the anti-G straining maneuver. Such environments of rapid-onset, highly-sustained +Gz cause severe emotional and physical stress in the pilots [4, 5]. Therefore, it is important for both physicians and aviation medicine researchers to be aware of any pathophysiological alterations caused by hypergravity exposure. Numerous studies on human exposure to +Gz have demonstrated various pathophysiological effects, including changes in cerebral and coronary blood flow, cardiovascular reflexes, and endocrine reactions [6–11].

In addition to a decrease in cerebral and coronary blood flow, hypergravity also leads to structural and functional alterations in the visceral organs, especially, the impairment of visceral circulation [12, 13]. A reduction in visceral blood flow may result from the combination of hypergravity-induced cardiovascular reflex responses, emotional stress causing sympathetic vasoconstriction, and the increased peripheral vascular resistance of visceral beds. Previous studies have shown that exposure to hypergravity leads to significantly decreased blood flow to the spleen, pancreas, liver, and kidneys, possibly to maintain sufficient blood flow to the brain and heart [12–14].

The gastrointestinal system is especially vulnerable to stress, as demonstrated by the stress-induced alterations in gastric acid secretion and intramucosal blood flow [15]. Although the effects of hypergravity exposure on cardiovascular and cerebral functions have been extensively studied [7–9, 13, 14], the potential pathophysiological effects on the gastrointestinal tract are unknown. Recently, we demonstrated a significant reduction in gastric secretory activity in rats subjected to hypergravity exposure [5]. To support this finding, we needed to identify the mechanism involved in hypergravity-induced gastric acid hyposecretion. Based on the results of hypergravity-induced increase in catecholamines produced by the adrenal glands and a decrease in gastric secretory activity after the stimulation of the sympathetic nervous system [16, 17], we hypothesized that hypergravity-induced gastric acid hyposecretion is related to the adrenal glands. To verify this hypothesis, we investigated the effect of bilateral adrenalectomy on gastric juice parameters and plasma gastrin level in hypergravity-exposed rats. In continuation of our previous study, we used the pylorus ligation technique to induce gastric acid hypersecretion [5, 18]. We measured gastric juice parameters, including volume, acidity, and pH, and plasma gastric level.

## RESULTS

### Effects of hypergravity exposure and adrenalectomy on gastric juice parameters

**Figure 1:**
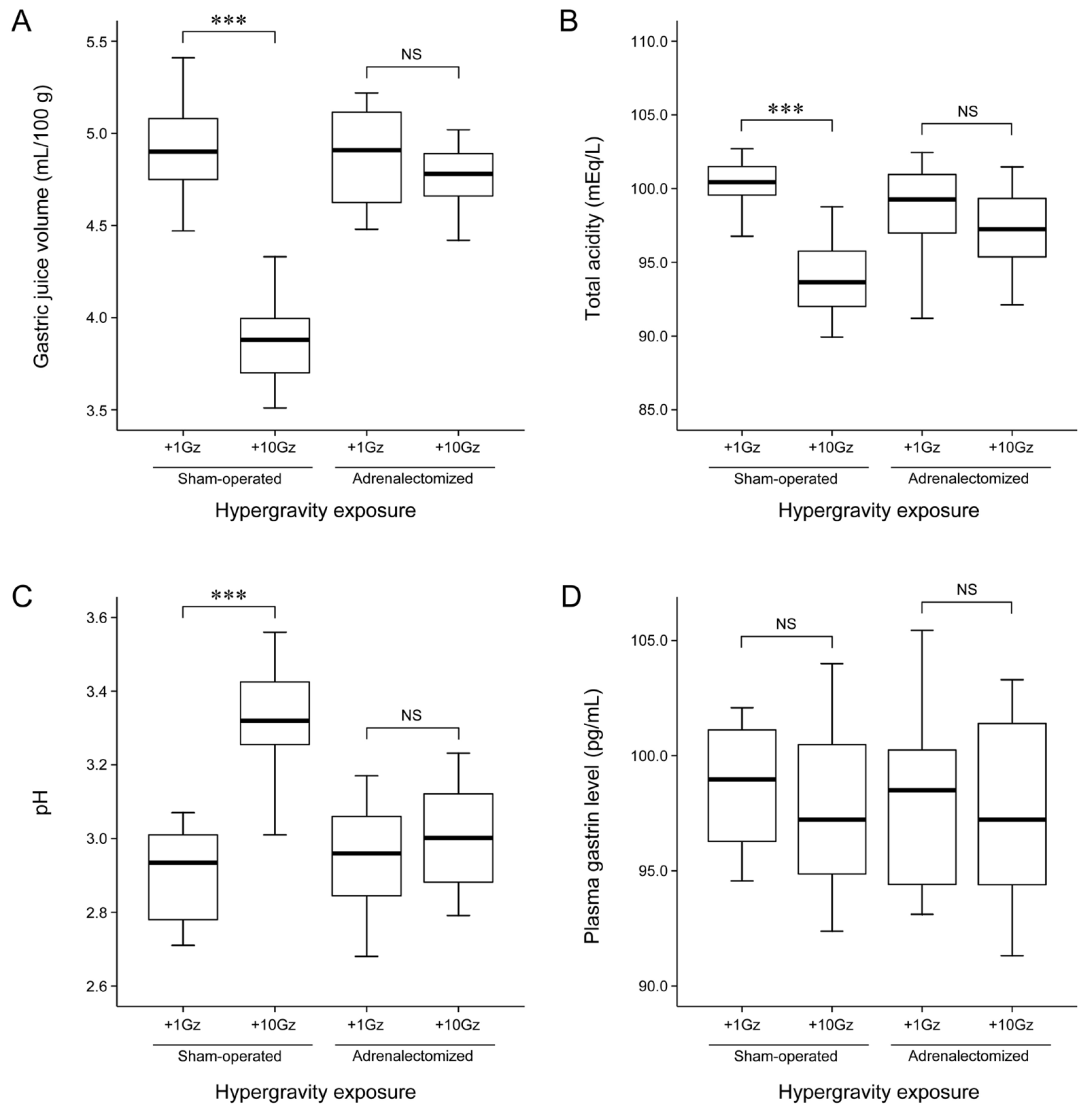
Effects of adrenalectomy on gastric juice parameters and plasma gastrin level in hypergravity-exposed rats Box and whisker diagram. A band inside each box indicates the median value of each group. In the sham-operated rats, acute exposure to hypergravity significantly decreased gastric juice volume (*P* < 0.001) and total acidity (*P* < 0.001) and increased gastric juice pH (*P* < 0.001). In contrast, in the adrenalectomized rats, there were no significant differences in the volume (*P* = 0.712), total acidity (*P* = 0.279), and pH (*P* = 1.000) of gastric juice between the hypergravity-exposed and nonexposed rats. In addition, acute exposure to hypergravity in the sham-operated rats did not significantly alter plasma gastrin level (*P* = 0.985). Also in the adrenalectomized rats, there was no significant difference in plasma gastrin level between the hypergravity-exposed and nonexposed rats (*P* = 0.990). ***P < 0.001; NS, not statistically significant.

First, we examined the influence of hypergravity exposure on gastric juice parameters. Differences in gastric juice parameters between the groups are shown in Figure 1. Acute exposure to hypergravity significantly altered gastric juice parameters. In sham-operated rats, the gastric juice volume of the hypergravity-exposed rats (3.87±0.26 mL/100 g) was significantly lower than that of the nonexposed rats (4.91±0.27 mL/100 g; *P* < 0.001; Figure 1A). Similarly, the total gastric juice acidity of the hypergravity-exposed rats (93.75±2.74 mEq/L) was significantly lower than that of the nonexposed rats (100.96±3.10 mEq/L; *P* < 0.001; Figure 1B). The gastric juice pH of the hypergravity-exposed rats (3.33±0.15) was significantly higher than that of the nonexposed rats (2.90±0.12; *P* < 0.001; Figure 1C). These results are in agreement with our previous data showing that hypergravity exposure significantly reduces the volume and acidity of gastric juice and elevates its pH [5].

Second, we evaluated the effect of bilateral adrenalectomy on gastric juice parameters in the hypergravity-exposed rats. In the adrenalectomized rats, acute exposure to hypergravity did not produce a significant decrease in the volume and acidity of gastric juice. The gastric juice volume of the adrenalectomized, hypergravity-exposed rats (4.77±0.22 mL/100 g) was not significantly different from that of the adrenalectomized, nonexposed rats (4.88±0.26 mL/100 g; *P* = 0.712; Figure 1A). The total gastric juice acidity of the hypergravity-exposed rats (96.48±2.73 mEq/L) was slightly lower than that of the nonexposed rats (98.59±3.07 mEq/L), but the difference was not statistically significant (*P* = 0.279; Figure 1B). The adrenalectomized rats did not show a significant elevation in gastric juice pH following hypergravity exposure, which was observed in the sham-operated rats. The gastric juice pH of the adrenalectomized, hypergravity-exposed rats (3.00±0.15) was not significantly different from that of the adrenalectomized, nonexposed rats (2.94±0.16; *P* = 1.000; Figure 1C). These results indicate that adrenalectomy produced a significant increase in gastric juice volume and acidity, suggesting that the adrenal glands are required for hypergravity-induced gastric acid hyposecretion in rats. The group means of gastric juice volume, total acidity, and pH are shown in Table 1.

**Table 1:** Effects of adrenalectomy on gastric juice parameters in hypergravity-exposed rats

Group	Mean gastric juice volume (mL/100 g)	Mean total acidity (mEq/L)	Mean pH
Sham-operated / +1Gz	4.91 ± 0.27	100.96 ± 3.10	2.90 ±0.12
Sham-operated / +10Gz	3.87 ± 0.26	93.75 ± 2.74	3.33 ± 0.15
Adrenalectomized / +1Gz	4.88 ± 0.26	98.59 ± 3.07	2.94 ± 0.16
Adrenalectomized / +10Gz	4.77 ± 0.22	97.48 ± 2.73	3.00 ± 0.15

### Effects of hypergravity exposure and adrenalectomy on plasma gastrin level

Consistent with our previous study [5], acute exposure to hypergravity did not significantly alter plasma gastrin level. In sham-operated rats, there was no significant difference in plasma gastrin level between the hypergravity-exposed (97.92±4.02 pg/mL) and nonexposed (98.50±2.55 pg/mL) rats (*P* = 0.985; Figure 1D). Similarly, in the adrenalectomized rats, the difference in plasma gastrin level between the hypergravity-exposed (97.86±3.79 pg/mL) and nonexposed (97.43±4.17 pg/mL) rats was not statistically significant (*P* = 0.990; Figure 1D). These results indicate that adrenalectomy has no effect on plasma gastrin level. The group means for plasma gastrin level are shown in Table 2.

**Table 2:** Effects of adrenalectomy on plasma gastrin level in hypergravity-exposed rats

Group	Mean plasma gastrin level (pg/mL)
Sham-operated / +1Gz	98.50 ±2.55
Sham-operated / +10Gz	97.92 ± 4.02
Adrenalectomized / +1Gz	97.86 ± 3.79
Adrenalectomized / +10Gz	97.43 ± 4.17

## DISCUSSION

Gastric acid aids digestion and kills bacteria through a chemical reaction by activating pepsin. Stress-induced activation of the sympathetic nervous system causes the inhibition of gastric secretory activity by stimulating catecholamine release from the adrenal glands. The resulting reduction of gastric acidity will significantly lower pepsin activity, which in turn undermines protein digestion. If proteins are not properly broken down, they can act as potentially pathogenic antigens in the human body. In severe cases, these proteins can cause gastroesophageal reflux and flatulence by increasing gastric acidity abnormally while overstaying in the stomach. Fighter pilots having gastrointestinal disorders, such as dyspepsia, abdominal discomfort, and/or abdominal pain as a result of abnormalities of gastric secretory activity, may experience significant difficulties in performing their task during flying [5].

In our previous study, we found that hypergravity exposure significantly reduced the volume and acidity of gastric juice, whereas, no significant difference was observed in plasma gastrin levels between the hypergravity-exposed and nonexposed rats [5]. These findings were confirmed in the present study. Acute exposure to hypergravity inhibits gastric acid secretion, but it is unlikely that the decreases in gastric juice volume and acidity due to hypergravity exposure were caused by gastrin. To the best of our knowledge, there has been no study on the mechanism that explains the effects of hypergravity exposure on gastric secretory activity. We hypothesized that acute exposure to hypergravity would stimulate the sympathetic nervous system and consequently, increase catecholamine release from the adrenal glands and inhibit gastric acid secretion. To verify this hypothesis, the effects of adrenalectomy on gastric acid parameters were investigated. Hypergravity-induced decreases in gastric acid volume and acidity that sham-operated rats demonstrated were not observed in the adrenalectomized, hypergravity-exposed rats. In other words, the hypergravity-induced changes in gastric acid parameters were not statistically significant in the adrenalectomized rats. These findings suggest that adrenalectomy abolishes the hypergravity-induced gastric acid hyposecretion and that the adrenal glands are involved in the hypergravity-induced gastric acid hyposecretion. Hypergravity-induced decrease in visceral blood flow and increases in plasma levels of epinephrine, norepinephrine, and corticosterone support the notion that high levels of catecholamine driven by the stimulation of the sympathetic nervous system may cause splanchnic vasoconstriction, which may slow blood flow to visceral organs including stomach [7, 11–14, 17]. In the present study, we speculated that hypergravity-induced reduction in gastric acid secretion may be due to reduction of blood flow to stomach. Moreover, we cannot rule out the possibility of decreased acid secretion that may result from gastric tissues being physically compressed when visceral organs shift toward the lower end of the body under the influence of hypergravity.

The association between gastric acid secretion and the adrenal gland in the human body can manifest differently, depending on pathophysiological conditions. Decreased gastric acidity and low incidence of chronic peptic ulcer in patients with adrenal insufficiency suggest the involvement of the adrenal glands in gastric secretory activity [19]. Similarly, when the adrenal steroids or adrenocorticotropic hormone were administered, gastric acidity and pepsin secretion increased, signifying the effects of the adrenal glands on the pathogenesis of peptic ulcer disease. On the contrary, multiple gastrointestinal ulcerations in the pyloric region of the stomach observed in patients with acute adrenal insufficiency imply that adrenal function and gastric secretory activity are not always in parallel [19]. Acute gastric ulcerative lesions occurring during absolute or relative adrenal insufficiency in humans could be reproduced experimentally in animals by severe stress following adrenalectomy [19]. This result is consistent with our findings showing increased gastric acid volume and acidity after adrenalectomy. A previous study reported that changes in gastric secretory activity due to acute adrenal insufficiency may be attributed to vascular stasis and cellular damage accompanying the state of dehydration and electrolyte imbalance [19]. Histopathologically, ulcerative lesions caused by acute adrenal insufficiency are multiple and superficial, whereas lesions resulting from adrenal replacement therapy are manifested as discrete and deep, chronic peptic ulcers and occur when the hormone is administered for a long time. Clinical judgments on changes in gastric pathophysiology behind adrenal hypo- and hyperfunction or excessive adrenal hormone administration should be based on alterations in the mucosal barrier, interference with tissue repair, and decreased tissue resistance secondary to vascular and metabolic disturbances, in addition to secretory activity.

In conclusion, we demonstrated that adrenalectomy abolished hypergravity-induced gastric acid hyposecretion, but did not influence gastrin release. These findings suggest that the adrenal glands are involved in hypergravity-induced gastric acid hyposecretion.

## MATERIALS AND METHODS

### Experimental animal

Male Sprague-Dawley rats, 10-11 wk of age and weighing 280-300 g, were fed standard laboratory rat chow, provided with free access to water, and maintained on a 12-h light-dark cycle under temperature and moisture levels controlled at 20-25°C and 40-45%, respectively. In order to avoid the effects of unfavorable factors including fear and stress, the rats were allowed to acclimatize to the rearing environment for 7 d. The Institutional Animal Care and Use Committee of the Republic of Korea Air Force Aerospace Medical Center (Cheongju, Chungcheongbuk-do, Republic of Korea) approved all experimental procedures involving the animals. All experimental procedures involving the animals were conducted in accordance with the Guide for Care and Use of Laboratory Animals published by the National Institutes of Health and the ethical guidelines of the International Association for the Study of Pain.

### Bilateral adrenalectomy

The rats either were adrenalectomized (n = 24) or underwent sham adrenalectomy (n = 21). Bilateral adrenalectomy was performed via a dorsal approach. A one-inch midline incision was made on the skin along the back. After moving the incision towards either side of the kidney, a small cut was made on the muscle posterior to the last rib. The bilateral kidneys were located and the adrenal glands excised. Sham adrenalectomy entailed the same procedure as adrenalectomy, except that the adrenal glands were grasped but not removed. After surgery, the drinking water of the adrenalectomized rats was replaced with physiological saline (0.9% sodium chloride). The rats were kept warm (25°C) in the laboratory for observation for 6 h after which they were transferred to the housing unit.

### Pylorus ligation and hypergravity exposure

Our previous study confirmed that pylorus ligation increases gastric secretory activity, and that acute hypergravity exposure negatively influences on the gastric acid secretory function [5]. In this study, we aimed at investigaing the restoring effect of adrenalectomy on the gastric secretory activity in the same setting as that of our previous study. Under light anesthesia, laparotomy was performed through a midline incision of approximately 3 cm. The pyloric region of the stomach was gently mobilized and occluded with a 4-0 silk ligature around the pyloric sphincter [5, 20]. The incision was then closed with 3-0 silk sutures. Thirteen adrenalectomized and 11 sham-operated rats were exposed to +10Gz three times for 3 min (onset rate, +1Gz/s) using a small-animal centrifuge. Each rat was placed into a cylindrical plastic restraint device that, when mounted in a centrifuge, allowed +Gz to be delivered along the rostrocaudal axis. After the rats were secured, the restraint device was clamped to the end of the centrifuge arm, which allows one degree of freedom, thereby ensuring that the net G field was perpendicular to the floor of the restraint device [5, 21–23]. The hypergravity-nonexposed (+1Gz) rats were placed in the centrifuge arm and underwent a similar process to the one described above, but they were not exposed to hypergravity. The behavior of the rats was monitored with a charge-coupled device camera throughout the centrifugation experiments. None of the rats died from the surgical procedure or hypergravity exposure.

### Measurement of the gastric juice parameters

Three hours after pylorus ligation, the rats were euthanized. The abdomen was opened, the stomach was removed, and the gastric content was collected and centrifuged at 8,000×*g* for 10 min at 25°C. The volume (mL/100 g), total acidity (mEq/L), and pH of the gastric juice were determined. The total acidity was determined by titration to pH 7.0 with 0.01 N sodium hydroxide using phenolphthalein as an indicator. The pH was measured using a digital pH meter.

### Measurement of plasma gastrin level

Just before stomach removal, blood was collected from the abdominal aorta. The heparinized blood was centrifuged at 3,000×*g* for 10 min and the plasma was kept at –20°C until analysis. The plasma level of gastrin was determined using a commercially available enzyme-linked immunosorbent assay kit (Abcam, Cambridge, UK).

### Statistical analysis

All values are provided as the mean ± standard error. Differences between the groups were assessed using one-way analysis of variance followed by Tukey's multiple range tests. Statistical analyses were conducted using PASW Statistics for Windows (version 18.0; Armonk, NY, USA). Statistical significance was defined as a *P* value less than 0.05.
